# Editorial: Computational methods in cardiac electrophysiology

**DOI:** 10.3389/fphys.2023.1231342

**Published:** 2023-06-21

**Authors:** Matthijs Cluitmans, Richard Walton, Gernot Plank

**Affiliations:** ^1^ Department of Cardiology, Cardiovascular Research Institute Maastricht, Maastricht University, Maastricht, Netherlands; ^2^ INSERM Institut de Rythmologie et Modélisation Cardiaque (IHU-Liryc), Pessac, Aquitaine, France; ^3^ Gottfried Schatz Research Center for Cellular Signaling, Metabolism and Aging, Medical University of Graz, Graz, Styria, Austria

**Keywords:** electrophysiolgy, arrhythmias (cardiac), technology, digital twin, personalized medicine, signal processing, methodology

## Introduction

Cardiac electrophysiology research increasingly relies on computational methods to connect experimental and clinical observations to understand underlying mechanisms. These methods process experimental data, such as optical mapping and body-surface potential mapping, and model biophysical processes, such as the behavior of electrical sources within the heart and the electrical potential fields linked to these. Signal processing from experiments and clinical recordings helps elucidate electrophysiological properties across various domains, while computational modeling offers a theoretical understanding. Patient-specific models increasingly help interpret observations and improve individual cardiac electrical behavior approximations. Consequently, advancements in computational methodologies are vital for gaining new insights into cardiac electrophysiology and arrhythmias.

**FIGURE 1:**
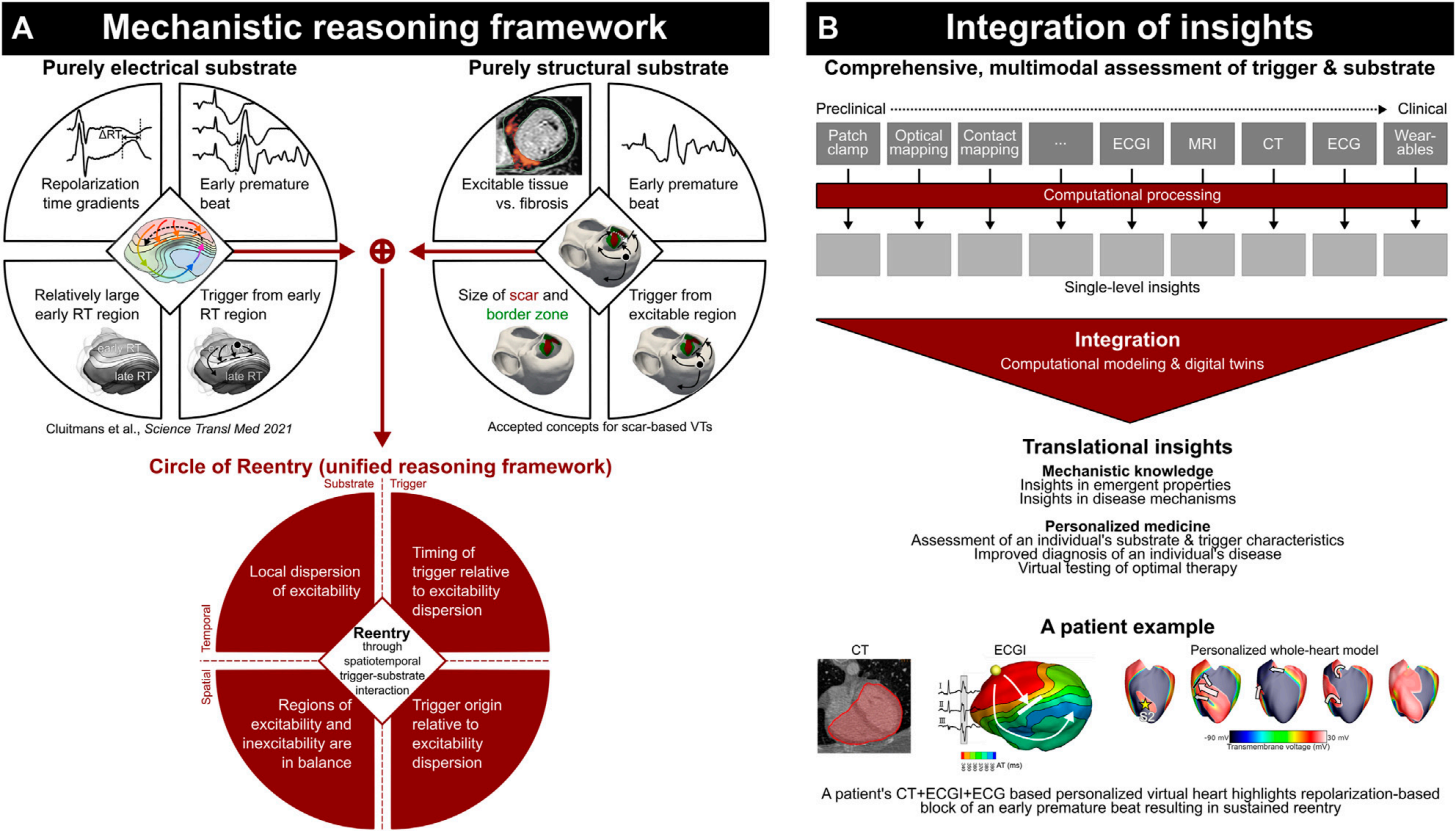
Current mechanistic reasoning frameworks highlight the complex spatiotemporal interaction of trigger and substrate **(A)**; computational modeling is essential to integrate detailed assessment of trigger and substrate characteristics to obtain translational insights **(B)**. Adapted from Cluitmans et al., 2023, licensed CC-BY-4.0.

Here, we review papers published in the Frontiers in Physiology Research Topic on “*Computational methods in cardiac electrophysiology*,” and share a perspective on the potential future impact of such technology (Figure 1).

## Body-surface potential mapping, electrocardiographic imaging, and optical mapping

Body-surface potential mapping (BSPM) may provide information beyond the 12-lead electrocardiogram (ECG), which is particularly relevant for extraction of therapy predictors in complex (chaotic) rhythms such as atrial fibrillation (AF). Zhong et al. used BSPM signals to predict AF recurrence after catheter ablation therapy. They combined single-time instant BSPMs with temporal-attention block, which allowed the training of a 3D convolutional neural network (3D-CNN) with BSPMs over time to predict AF recurrence. The advantage of using BSPM without performing noninvasive inverse mapping techniques (called electrocardiographic imaging, ECGI) is that it avoids ECGI’s particular intricacies and pitfalls. Melgaard et al. have performed such inverse mapping but have limited it to a simplified, potentially more stable, approach. They restrict their inverse technique to the 12-lead ECG and a generic (non-personalized) geometry. Their method was able to noninvasively localize the latest electrically activated region in patients with LBBB, which is relevant for lead placement during CRT implantation. Although the use of generic geometries forfeits the need for imaging in patients, it likely affects the accuracy with which abnormalities can be localized in the heart. This was also studied by Molero et al., who investigated the effect of the density of personalized digitized torso meshes in patients with AF undergoing ECGI. They found that including the exact positions of the electrodes on the patient’s torso directly in the mesh (thus matching electrodes with mesh nodes) drastically reduces the need for high-density meshes. Their findings suggest that meshes primarily composed of electrode positions may contain sufficient geometric detail for accurate inverse reconstructions (if sufficient electrodes are present).

In two companion papers, Meng et al. introduced a novel formulation of ECGI and then applied this method to the less-studied intracardiac approach. First, the method of fundamental solutions (MFS) was employed to map intracardiac (catheter-based) signals to the endocardium of the heart. MFS is a meshless ECGI approach that has been applied to inverse torso-heart mapping, but not yet to inverse catheter-heart (intracardiac) mapping. They studied the intracardiac MFS approach and found that it outperforms traditional (mesh-based) approaches and is theoretically simpler to set up. Subsequent application of this method in patients with AF showed that it is a feasible mapping method, but catheters must be large enough to capture features of complex rhythms (Meng S. et al.).

Combined, these papers add important insights to the field: For some applications, BSPM or simplified ECGI approaches (based on the 12-lead ECG) may be sufficient; and for more complex applications such as intracardiac mapping, meshless approaches may be better suited than the traditional mesh-based approaches with more complex requirements. Sometimes, simpler is better.

With the advent of telehealth, a robust quality assessment of input data from (wearable) sensors is essential. Castiglioni et al. used cepstral analysis, a method to identify the periodicity of a signal, for single-lead electrocardiograms to quantify the quality of the recording. Even if multiple electrodes are available, defining the most informative metrics remains an ongoing process, as illustrated by Kappel et al., who assessed three quantitative indices to predict whether a uniform ablation strategy resulted in AF termination (Kappel et al.).

Optical mapping plays a major role in unraveling arrhythmia mechanisms in experimental investigations. Such mechanisms may be partially based on complex interactions between transmembrane voltage and intracellular calcium. Uzelac et al. developed a method that allows simultaneous recording of these quantities in a single-camera optical mapping setup. This allows the quantitative characterization of their dynamic interactions that play a role in arrhythmogenesis at the tissue level. Among others, dynamics may be the result of inflammation, which was studied with computational models to begin unraveling the underlying complex interactions by Bi et al.

## Tissue modeling, organ modeling, and digital twins

Traditionally, computational models have been indispensable in experimental studies to facilitate more accurate analysis and to infer mechanisms underlying cardiac function. Driven by recent methodological advances, cardiac modeling has also begun to appear in clinical applications as a means of aiding in diagnosis and stratification, or—by exploiting their mechanistic nature that allows to predict therapy outcomes—for the optimal planning of therapies. All of these application scenarios pose different challenges, many of which are addressed in this Research Topic.

Overall, cardiac modeling benefits from methodological advances leading to improved robustness, accuracy and numerical stability (Barral et al.). It also profits from more accurate biophysical representation of mechanisms, such as electro-mechanical force generation at the cellular level (Bartolucci et al.) or of atrial electrophysiology in experimentally important porcine models (Peris-Yagüe et al.). Models also play a pivotal role in gaining insight into the relationship between key pathological processes in cardiac diseases such as myocardial fibrosis and their reflection in the most important observable physical measurements, that is, intracardiac electrograms. The computational methodology for best representing the impact of fibrosis in models on electrograms was comprehensively reviewed by (Sánchez and Loewe).

At the forefront of cardiac modeling research is the development of methodologies for generating anatomically accurate and physiologically detailed computational models calibrated to patient data at an individual level. Such personalized models represent data acquired from individuals with high fidelity, or are statistically representative of a group of patients. Such digital twin models or virtual cohorts are now gaining importance in clinical applications, in the medical device industry as well as in regulatory policy. Furthering these arguably most advanced models of cardiac function to deliver on these high promises relies on improving several critical aspects. Most importantly, model calibration must be achieved with high fidelity by comparing to clinically observable data. These calibration processes must be streamlined and automated to create digital twins with sufficient reliability within feasible timeframes. Gillette et al. reported the first biophysical whole-heart electrophysiology model that can be executed with real-time performance, and is able to match the ECG of the modelled subject using a topologically and physically detailed model of the entire cardiac conduction system. Beetz et al. proposed a novel data-driven approach to investigate physiological patterns linked to electrophysiological activity and mechanical deformation (Beetz et al.). These vary considerably between individual patients and across cardiovascular diseases. They developed a multi-domain variational autoencoder network that integrates electrocardiogram and MRI-based 3D anatomy data into a unified model. Demonstrating high fidelity reconstruction and generation of realistic virtual populations, their approach enhances cardiovascular disease classification and supports the creation of accurate computational models, capturing disease and patient variability. Doste et al. proposed utilizing cardiac models for accurately and non-invasively determining the site of origin of ectopic beats in outflow tract arrhythmias prior to ablation therapy, to improve intervention outcomes. They enriched the training data with simulation-based synthetic data to train a machine-learning classification model. Their study demonstrated that simulated data are pivotal for enhancing training classification algorithms to achieve sufficiently accurate localization of the sites of origin.

Personalized computational methodology is ideally positioned—and perhaps critical—to assess an individual’s true risk for arrhythmias, as it is increasingly recognized that simplified concepts such as “wave length,” “scar volume,” and “reduced ejection fraction” are insufficient to accurately assess this. This is particularly true when addressing both electrical and structural abnormalities, as can be studied using the recently introduced unifying “Circle of Reentry” (Cluitmans et al., 2023), Figure 1A. Although such reasoning frameworks may be all-encompassing, they are dependent on a complex spatiotemporal interaction of their elements. This necessitates computational assessment not just to process the complex data at the level of each single modality, but also to integrate findings to understand emergent behavior and arrive at an accurate risk assessment of an individual’s heart characteristics (Figure 1B).

Artificial intelligence (AI), although relatively unaddressed in this collection of papers, is expected to significantly change our scientific understanding (Krenn et al., 2022). Although its ‘black-box’ approach may initially seem unsuitable to obtain new insights, it can uncover patterns and may be well suited to explore new or unstructured data. Even when the biophysics of a field are relatively well-understood—as is arguably the case for cardiac electrophysiology—an interaction between “fuzzy” AI and ‘exact’ biophysics may yield new insights (Cluitmans, 2023). And AI in Digital Twins may help to augment data that is required for personalization but may not be directly available in a particular individual.

## Conclusion

In conclusion, the papers presented in this Research Topic on “*Computational methods in cardiac electrophysiology*” collectively contribute to advancements in computational methods that can transform the understanding, analysis, and treatment of cardiac arrhythmias. By focusing on the development of robust and accurate models, as well as innovative approaches to personalize patient care, these methodologies pave the way for better clinical applicability and predictive outcomes. The integration of artificial intelligence and machine learning techniques, which have seen rapid growth and adoption in recent years and months, is poised to propel the field forward, enabling deeper insights and more effective treatment strategies. Overall, the synergy of computational electrophysiology, AI, and experimental and clinical data holds great promise for improved diagnostic accuracy, patient-specific therapeutic planning, and an enhanced understanding of complex cardiac interactions, ultimately contributing to better patient outcomes and quality of life.
